# 917 Multidisciplinary Burn Outreach Committee Improves Burn Prevention and Staff Engagement

**DOI:** 10.1093/jbcr/iraf019.448

**Published:** 2025-04-01

**Authors:** Anna Ehoff, Rosie Rufo, Crystal Grijalva, Francesca Ferrigno, Nicole Kopari, Catrina Cullen

**Affiliations:** University of California San Francisco, Fresno Leon S. Peters Burn Center; University of California San Francisco, Fresno Leon S. Peters Burn Center; University of California San Francisco, Fresno Leon S. Peters Burn Center; University of California San Francisco, Fresno Leon S. Peters Burn Center; University of California San Francisco, Fresno Leon S. Peters Burn Center; University of California San Francisco, Fresno Leon S. Peters Burn Center

## Abstract

**Introduction:**

Per the 2024 ABA re-verification requirements, a burn center must have a burn prevention program active in the community to promote prevention and awareness. In prior years, we utilized a burn outreach coordinator. Historically, a registered nurse with robust burn experience occupied the position. However, the aftermath of the COVID-19 pandemic resulted in high staff turnover and a low applicant pool for this position. Staff engagement averaged 3 participants per community event. Overall participation of the multidisciplinary burn team was approximately 15%. The burn center participated in, on average, 1 event per month for the year 2023. The burn center sought a creative solution to reintegrate burn prevention and awareness in the community and increase staff engagement.

**Methods:**

A multidisciplinary burn outreach committee was developed in December 2023. The foundational burn staff members comprised the director of critical care, nursing burn manager, burn registrar, and administrative secretary. Additional foundational members included ancillary staff members such as social work and child life specialists. To support data capture for staff engagement in burn outreach events, we collected burn staff attendance and number of events from December 2023 to September 2024.

**Results:**

The multidisciplinary burn outreach committee has allowed us to use diverse perspectives and expertise to improve team collaboration. Since establishing the committee, we have successfully held monthly community outreach events. During the review period, we found the median staff attendance to be 6. There were 24 outreach events, with over 50% of our staff volunteering for at least one event from December 2023 to September 2024.

**Conclusions:**

The development of the multidisciplinary burn outreach committee assisted with revitalizing our presence in the community to promote burn prevention and awareness. In addition, this committee has allowed us to optimize each discipline’s skill set, which is critical for applying a holistic approach to burn prevention and burn awareness in a community with a complex patient population.

**Applicability of Research to Practice:**

As a trauma center caring for burn patients, ABA guidelines require an organized and coordinated multidisciplinary team effort during hospitalization. This committee is optimizing an interdisciplinary team for burn outreach to educate the community about burn prevention and burn awareness.

**Funding for the Study:**

N/A

